# Identification of homogeneously staining regions by G-banding and chromosome microdissection, and FISH marker selection using human Alu sequence primers in a scleractinian coral *Coelastrea aspera* Verrill, 1866 (Cnidaria)

**DOI:** 10.3897/CompCytogen.v10i1.5699

**Published:** 2016-01-22

**Authors:** Takahiro Taguchi, Satoshi Kubota, Takuma Mezaki, Erika Tagami, Satoko Sekida, Shu Nakachi, Kazuo Okuda, Akira Tominaga

**Affiliations:** 1 Division of Human Health and Medical Science, Graduate School of Kuroshio Science, Kochi University, Nankoku, Kochi 783-8505, Japan; 2 Kuroshio Biological Research Foundation, Otsuki, Hata County, Kochi 788-0333, Japan; 3 Department of Molecular & Cellular Biology, Kochi Medical School, Kochi University; 4 Division of Marine Bioresources, Graduate School of Kuroshio Science, Kochi University, 2-5-1 Akebono-cho, Kochi 780-8520, Japan

**Keywords:** Coral, karyotype, hsr, FISH, chromosome microdissection, Alu repeats

## Abstract

Karyotype analysis was performed on the scleractinian coral *Coelastrea aspera* Verrill, 1866, commonly found along temperate coasts in Japan (30–35°N) and in coastal waters in the Indian and Pacific oceans. G-banding of *Coelastrea aspera* was successfully performed, although the banding pattern was not as clear as that in mammals. The karyogram clearly revealed that this coral had a homogeneously staining region (hsr) in chromosome 11. This hsr consisted of ribosomal RNA (rRNA) related genes, which was demonstrated by fluorescence in situ hybridization (FISH) with probes generated using 28S ribosomal DNA (rDNA) primers and those generated through chromosome microdissection. In addition, we conducted silver-stained nucleolus organizer region (Ag-NOR) analysis and found Ag depositions in the interphase nuclei but not on rRNA gene loci and hsr(s) in the mitotic stage. The hsr of this coral was observed in approximately 50% of the metaphase spreads analyzed. This may explain the diversity of coral rDNA based on the molecular study of sequence analysis. Furthermore, it was discovered that human telomere and Alu repeated sequences were present in this *Coelastrea aspera*. Probes derived from human Alu sequences are expected to play an important role in the classification of corals. Overall, our data can be of great value in discriminating among scleractinian coral species and understanding their genetics, including chromosomal evolution.

homogeneously staining region

ribosomal RNA

fluorescence in situ hybridization

ribosomal DNA

silver-stained nucleolus organizer region

## Introduction

Species of the genus *Coelastrea* Verrill, 1866 belong to the family Merulinidae (Huang et al. 2014) and form massive colonies, usually spherical or elongated, with well-developed pale lobes. *Coelastrea aspera* Verrill, 1866 used in the present study is found in the Red Sea and Gulf of Aden, the southwest and northern Indian Ocean, the central Indo-Pacific, Australia, Southeast Asia, Japan and the East China Sea, the West Pacific, and the Central Pacific (DeVantier et al. 2014).

In scleractinian corals, the available chromosomal data, including their karyotypes and gene loci on chromosomes, has been limited, although many studies of other aspects of their biology such as ecology and physiology have been reported. Chromosomal evolution in corals occurs in closely related taxa within and at the species level (Kenyon 1997). The establishment of each karyotype among many coral species will help promote genetic research and coral genome projects (Shinzato et al. 2011).

In humans, a karyotype is usually established using the G-banding pattern shown on chromosomes (Seabright 1973). However, it has been difficult to get G-banding patterns in invertebrates, including scleractinian corals. Their G-banding pattern may facilitate the establishment of their karyotypes. Thus far, we have reported the karyotypes of two scleractinian corals belonging to the genera *Acropora* and *Echinophyllia* by molecular cytogenetic techniques (Taguchi et al. 2013, 2014). We found a characteristic chromosome with a homogeneously staining region (hsr)-like structure consisting of ribosomal RNA (rRNA) genes that were demonstrated by fluorescence in situ hybridization (FISH) in both corals (Taguchi et al. 2013, 2014). An hsr was originally found in mammalian cells as the result of a huge number of gene amplifications through drug-selection using methotrexate (Biedler and Spengler 1976). An hsr is a chromosomal segment(s) with various lengths and uniform staining intensity after G banding and sometimes found in mammalian cancer cells. This type of aberration has been known as an amplification of oncogenes (Takaoka et al. 2012). It is important to investigate if the presence of this hsr-like structure is a common phenomenon in coral chromosomes. Furthermore, there may be a possibility that the hsr-like structure involves not only rDNA but other specific sequences.

Obtaining FISH markers is not only profitable in helping to establish karyotypes but also in comparing syntenic relations among coral species. In our previous study (Taguchi et al. 2013), we selected a probe that was obtained from polymerase chain reaction (PCR) using the primers of human satellite DNA sequences and demonstrated some specific signals on the chromosomes of the coral *Echinophyllia aspera*, i.e., the fact that coral DNA shared common sequences with humans, such as the telomere consensus sequence (TTAGGG)n (Zielke and Bodnar 2010) and satellite III, which contains the consensus sequence (TTCCA)n (Taguchi et al. 2013). These findings prompted us to screen other repeated DNAs from corals, which may share sequences with those of humans.

In this study, we analyzed *Coelastrea aspera* chromosomes using not only a conventional G-banding method but also molecular cytogenetic techniques. We successfully identified the hsr of *Coelastrea aspera* chromosome 11 using trypsin-treated G-banding, followed by karyotyping. Following that, we conducted chromosome microdissection (CMD) to regenerate DNA sequences consisting of the hsr to see if this involved not only rDNA but also some other specific sequences. Then, we applied CMD technique. Microdissected DNA was amplified by PCR and used as a painting probe of hsr. Moreover, to see the nature of the rRNA gene on the chromosomes of *Coelastrea aspera*, we performed silver-stained nucleolus organizer region (Ag-NOR) analysis (Bloom and Goodpasture 1976, Trerè et al. 2000) and found that interphase nuclei appeared as black dots combined with silver grains, but not on rRNA gene loci including hsrs on metaphase chromosomes. Furthermore, we revealed by FISH analysis that sequences homologous to human Alu repeats are found in *Coelastrea aspera* chromosomes and we showed that these DNA markers for FISH may become good tools for classifying corals.

## Materials and methods

### Coral collection

**Figure 1.**
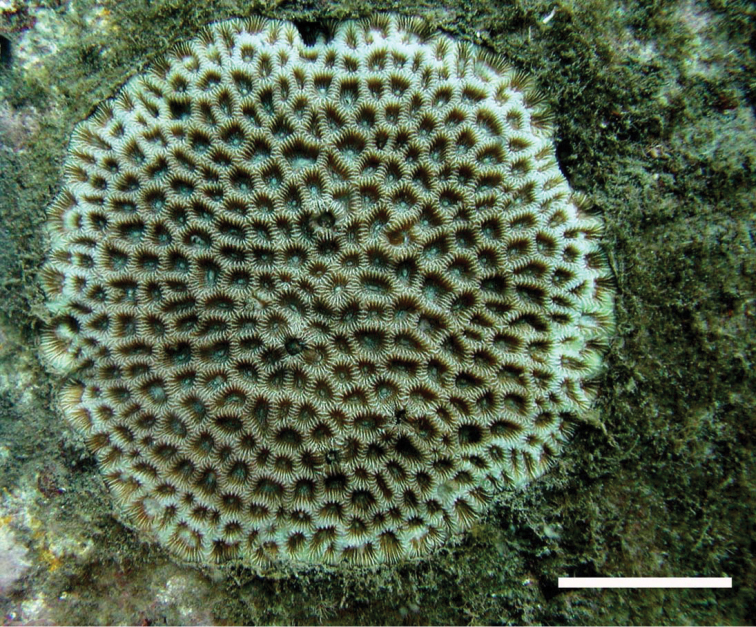
Appearance of *Coelastrea aspera* in the sea. Scale bar: 3 cm.

*Coelastrea aspera* is hermaphroditic; that is during spawning, eggs and sperm packed together into discreet bundles are released from the mouths of fertile polyps. The gametes of *Coelastrea aspera* were collected at Nishidomari (32°46'N; 132°43'E), in Kochi Prefecture, Japan (Fig. 1). The release of gamete bundles was observed between 8:45 pm and 9:15 pm on July 29, 2013; these bundles were collected in the field using plastic cups placed over part of the colonies during spawning. After collection, egg-sperm bundles were broken apart, and then eggs and fertilized eggs were rinsed using 0.2 μm filtered seawater (ADVANTEC cartridge filter; Advantec Toyo corp., Tokyo, Japan) to remove external contaminants.

### Chromosome preparations and G-banding

We have previously reported the method for making coral chromosome preparations (Taguchi et al. 2013, 2014). In brief, 9–12 h after artificial fertilization, the embryos were treated with 0.005% (v/v) colchicine (Sigma, St. Lous, MO, USA) followed by a hypotonic solution to spread the chromosomes and then fixed with a fresh mixture of absolute methanol and glacial acetic acid (3:1). Fixed embryos were soaked in diethyl ether overnight to remove intracellular lipids and returned to the fixative. Twenty to fifty embryos were cut into several pieces for which a fine needle was used to tear them apart into their constituent cells. Suspensions containing these small pieces of embryos were transferred into a 1.5 mL tube filled with fixative. The tube was centrifuged at 2000 × *g* for 2 min and then the pellet was re-suspended in 0.5 ml of fresh fixative. One drop containing fragments of embryos and isolated cells was placed on a clean slide and then air-dried to spread the chromosomes. For G-banding, air-dried slides with metaphase spreads were treated with 0.025% trypsin solution for 1 min, and then stained with Giemsa solution (Wako, Osaka, Japan) that was diluted to 5% with 0.06 M phosphate buffer (pH 6.8) before use. Human metaphase cells were harvested from male normal lymphocytes, and slides were prepared according to standard methods (Taguchi et al. 1993).

### Ag-NOR staining

Metaphase spreads on a slide were stained with silver solution (50% AgNO_3_ solution at 37 °C for 1–5 h), which binds to the nucleolus organizing regions (NOR), i.e., the secondary constrictions (stalks) of acrocentric chromosomes in the case of humans (Bloom and Goodpasture 1976).

### DNA extraction

Coral DNA from *Coelastrea aspera* embryos (approximately 200–300) or sperm (approximately 0.1 mL) was extracted using a Wizard genomic DNA purification kit purchased from Promega corporation (Madison, WI, USA) according to the manufacturer’s instructions.

### Chromosome microdissection of an hsr portion of chromosome 11

Five hsrs were scraped using a chromosome microdissection (CMD) technique as previously described (Taguchi et al. 2003). In brief, a glass needle with a diameter of about 2 μm was prepared from a glass capillary (GD-1, Narishige, Tokyo, Japan) using a pipette puller, PC-10 (Narishige). CMD was then performed using an inverted microscope (Olympus, Tokyo, Japan) with a glass needle attached to a mechanical micromanipulator, Eppendorf 5171 (Hamburg, Germany). Five microdissected hsr fragments were placed in a 0.5 mL tube, and then a 20 μL aliquot of digestion buffer containing 0.5 mg/mL proteinase K in 100 mmol/L Tris-HCl (pH 8.0) was added. Degenerate oligonucleotide primed polymerase chain reaction (DOP-PCR) was used to universally amplify the microdissected hsr DNA in a thermal cycler, WK-0518 (Wako, Osaka, Japan). The procedure used was essentially the same as that of Weber et al. (1998). PCR was conducted in a final volume of 50 μL containing 4 μL 25 mmol/L MgCl_2_, 5 μL 10× PCR buffer (500 mmol/L KCl, 100 mmol/L Tris-HCl, pH 8.0), 2 μL 5 mmol/L deoxynucleotide triphosphate, 5 μL 17 mmol/L primer 6 MW (5´-CCGACTCGAGNNNNNNATGTGG-3´, with N = A, C, G, and T), 0.5 μL of (2.5 U) Taq polymerase (Takara, Japan), and 20 μL of microdissected DNA in 100 mmol/L Tris-HCl, pH 8.0. The PCR conditions used were as follows: 10 min at 93 °C, followed by 10 cycles, each of 1 min duration at 94 °C, 1.3 min at 30 °C, 3 min transition at 30–72 °C, and 3 min extension at 72 °C; subsequently, 35 cycles, each of 1 min duration at 94 °C, 1 min at 62 °C, 3 min at 72 °C, and an additional 1 s/cycle to the extension step, and a final extension of 10 min.

### Generation of probes of rRNA genes and human Alu DNA sequences

**Figure 2.**
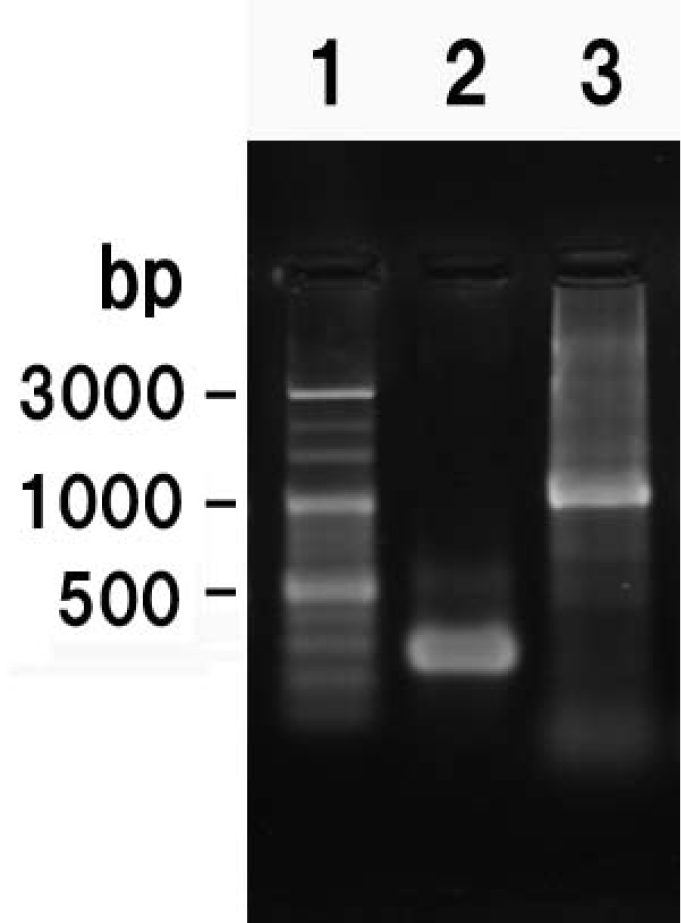
Electrophoresis of PCR products from the partial rDNA and the sequence generated by human Alu primers. **1** 100bp marker **2** 28S rDNA **3** Alu.

The probes for the rRNA genes and Alu-derived DNAs were obtained from PCR products using the primers described by Chen et al. (2000) and Watson et al. (1992), respectively. The gene encoding nuclear partial 28S rDNA for Acropora and human Alu-derived DNAs was amplified from *Coelastrea aspera* embryo DNA by a polymerase chain reaction (PCR), using 28s rDNA forward 5´-GGCGACCCGCTGAATTCAAGCATAT-3´ and reverse 5´-GCTTTGGGCTGCAGTCCCAAGCAACCCACTC-3´ primers (Chen et al. 2000). For the human Alu DNA sequence, the forward 5´- AACGTCACTCGGCTCTA-3´ and the reverse 5´- TTGCAGTGAGCCGAGAT -3´ primers were used (Watson et al. 1992). PCR was performed using a thermal cycler (WK-0518, Wako, Osaka, Japan). The PCR reaction conditions for 28S rDNA were as follows: pre-heating for 2 min at 95 °C, followed by 4 cycles at 94 °C (30 s), 60 °C (1 min) and 68 °C (3.5 min), and 30 cycles at 94 °C (30 s), 56 °C (30 s), and 72 °C (1 min), with a final extension at 72 °C for 10 min. The PCR reaction conditions for human Alu DNA was as follows: pre-heating for 2 min at 98 °C, followed by 30 cycles at 98 °C (10 s), 55 °C (30 s), and 72 °C (1 min). Figure 2 shows the electrophoresis image of PCR product obtained by the 28S rDNA and human Alu primers. The amplified bands on the gel (200–300 bp band for 28S rDNA; 1000 bp band for Alu; Fig. 2) were cut out and purified using the QIAquick PCR purification kit (Quiagen) according to the manufacturer’s instructions.

### Fish

A human digoxigenin-labeled telomere probe was purchased from Appligene Oncor (Lifescreen, Watford, UK). Random prime labeling of the probe DNA from PCR products was performed with fluorescein-12-dUTP (F-dUTP) or cyanine-3-dUTP (Cy3-dUTP) in accordance with the kit protocol (Invitrogen, Tokyo, Japan). FISH was conducted as previously reported (Takaoka et al. 2012), with slight modifications. In brief, the metaphase preparations were denatured in 70% formamide/2× saline-sodium citrate at 73 °C for 3 min. Then 1 μl of probe was mixed with 10 μl of hybridization solution (H7782, Sigma, Japan) and denatured at 80 °C for 10 min. Hybridization of the probes was performed at 37 °C in the CO_2_ incubator for 12–15 h, followed by post-hybridization washes, DAPI (4’,6-diamidino-2-phenylindole) counterstaining and visualization of the probes under a fluorescence microscope (Olympus BX50).

### Image acquisition and processing

The slides were examined with an Olympus BX-50 fluorescence microscope. Images of suitable metaphase spreads and interphase nuclei were acquired on an Olympus DP70 microscope workstation equipped with a cooled charge-coupled device and FISH analysis software. The miller units used for each fluorescence light (FITC, Cy-3 and DAPI) were U-NIBA, U-MWU, and U-MWIB (Olympus), respectively.

## Results

### The diploid karyotype in *Coelastrea aspera*

**Figure 3.**
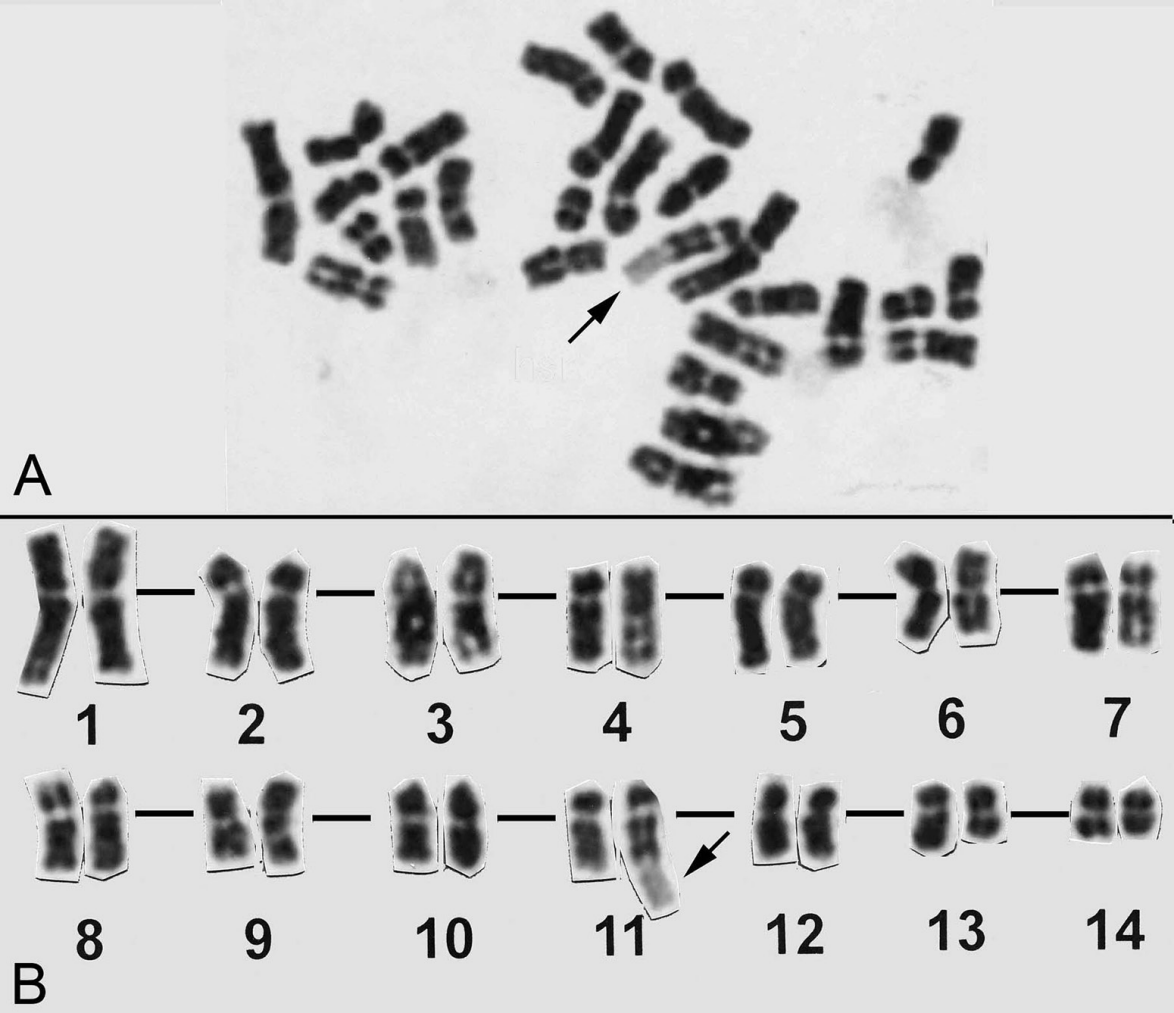
A G-banded metaphase spread (**A**) and its karyogram (**B**). Both stained with Giemsa. Arrows indicate hsrs.

A survey of 50 metaphase plates of *Coelastrea aspera* identified a complement of 28 chromosomes. Then we tried to make a conventional trypsin G-banding (Fig. 3A) and eight metaphase cells were karyotyped. Consequently, an hsr was identified in the long arm of chromosome 11 (Fig. 3B). During the survey of G-banded metaphase spreads, 31 metaphase spreads with an hsr were found in 60 cells examined (about 50%). A tentative karyogram was shown in Figure 3B. In this karyogram, chromosomes were arranged in decreasing order of chromosome length from 1 to 14. While chromosome 11 was easily identified due to the hsr and the location of its rRNA genes, other chromosomes were not so easy to distinguish precisely because of the poor G-banding pattern and similarities in chromosome length and centromere location. The karyogram consisted of thirteen submetacentric (pairs 1-13) and one metacentric (pairs 14) chromosomes (Fig. 3B).

### FISH mapping of rRNA genes and human telomere sequences, and Ag-NOR staining

**Figure 4.**
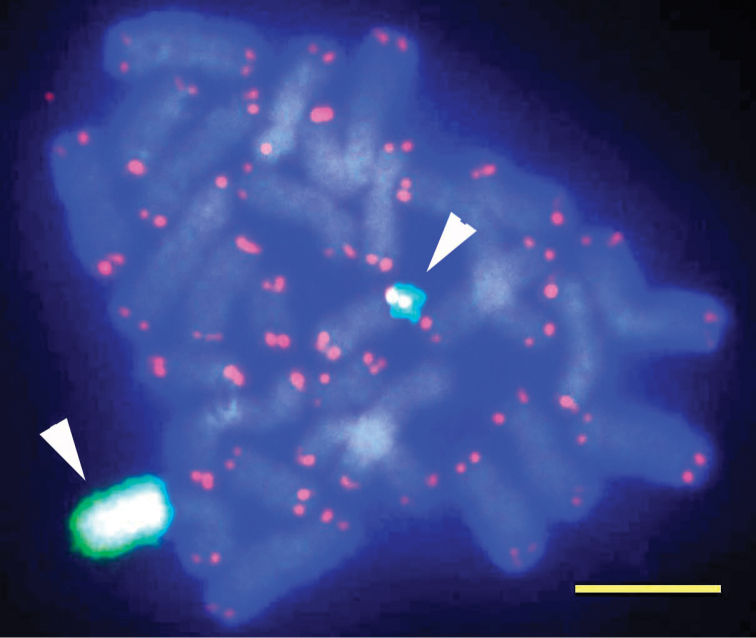
A dual color FISH image of *Coelastrea aspera* obtained by a probe generated by PCR using rRNA gene primers for 28S (green) and human telomere probe (red). Arrowheads indicate rRNA genes loci on one homologous chromosome 11 (one has an hsr with a long and large green signal). Scale bar: 5 μm.

**Figure 5.**
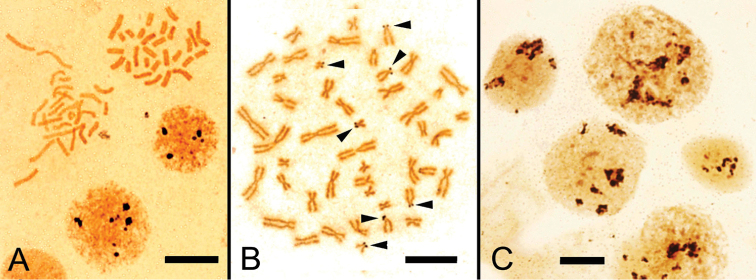
Ag-NOR staining. **A** Interphase nuclei and metaphases of *Coelastrea aspera*. Note that five to seven dark domains on interphase nuclei were seen but did not seem to appear on two metaphases **B** The human metaphase spread with black dots on the seven acrocentric chromosomes (arrowheads) **C** Human interphase nuclei. Scale bar: 10 μm.

Localizations of the rRNA genes by FISH are shown in Figure 4. The rRNA gene loci are mapped on one pair of homologous chromosome 11. Figure 4 shows the extra-large yellowish-green domain (left) with an hsr as well as small signals (right) in the metaphase spreads. The rate of metaphase spreads with an hsr was more than 50% (31/60). FISH analysis revealed that the human telomeric probe (TTAGGG)n hybridized intensely to the telomeres (red fluorescence) of all *Coelastrea aspera* chromosomes, suggesting that the telomere sequences of this coral may be identical to those of humans (Fig. 4). Ag-NOR staining showed five to eight dark dots on interphase nuclei, but no dot seemed to be seen on any chromosomes of *Coelastrea aspera* (Fig. 5A). The human metaphase spread, which was positive, was stained with AgNO_3_ at the same time as a control (Fig. 5B, C).

### CMD and FISH

**Figure 6.**
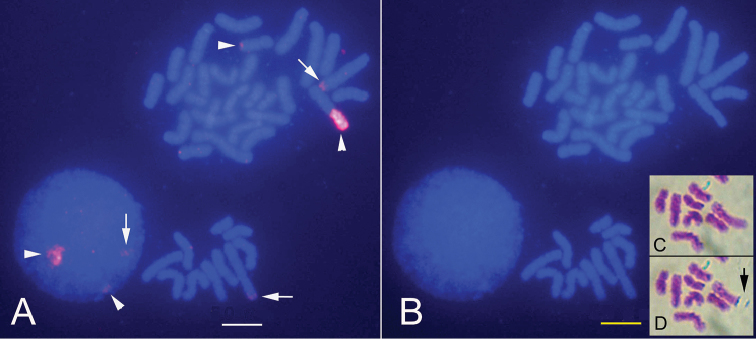
FISH signal by the probe from the microdissection-generated DNA probe of *Coelastrea aspera*’s hsr. **A** Metaphase cells with a distinct signal on the hsr (an arrowhead). Note that the hsr-derived probe hybridized not only with rRNA gene (arrowheads) but also with terminal ends of other chromosomes (arrows) in both full (2n=28, upper) and partial (lower) metaphases **B** The same cells were stained with DAPI. Chromosome microdissection of the hsr in chromosome 11 **C** Before microdissection of the hsr **D** After scraping of the hsr (an arrow). Scale bar: 5 μm.

Five hsr portions were scraped from five chromosome 11 by a glass needle (Fig. 6C, D). DOP-PCR products from these five microdissected chromosomes were observed as smear electrophoretic bands of DNA with a range from 200 to 900 bp on a 2% agarose gel, with most fragments being concentrated between around 400 and 600 bp. Screening with direct PCR probes by FISH demonstrated that the product had a high specificity not only on the hsr region of chromosome 11 but also on the terminal region of another chromosome (probably chromosome 2, Fig. 6A; arrows). FISH signals were also detected in an interphase nucleus. This microdissected DNA mainly consists of rDNA and may also contain some other sequences.

### Detection of human Alu sequence by FISH

**Figure 7.**
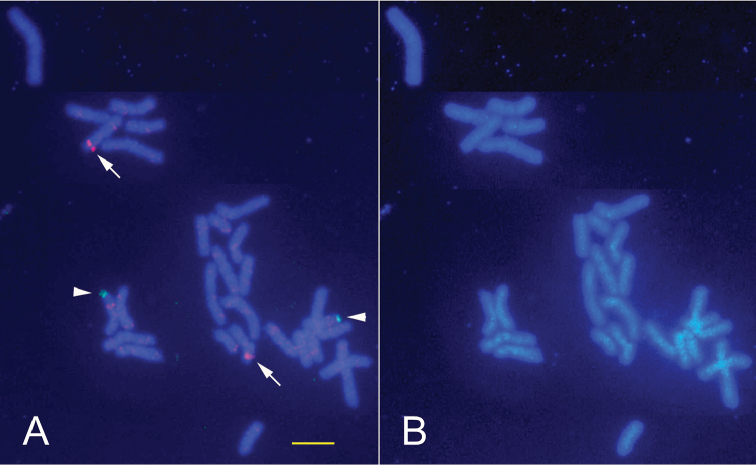
Dual FISH image with human Alu-derived and rRNA gene probes. **A** Red FISH signals by human Alu-repetitious DNA-derived probe were seen on two homologous chromosomes (arrowheads). Green signals by 28S rRNA genes (arrows). In this cell, no hsr was seen **B** The same chromosomes stained with DAPI. Scale bar: 5 μm.

Figure 7A shows four red signals on the FISH image of *Coelastrea aspera* obtained by the probe generated from the human Alu sequence primers. However, there were many scattered small faint red signals, suggesting that other Alu sequences were present. Two distinct reddish signals are seen on each telomeric region of two chromosomes of *Coelastrea aspera* (Fig. 7A).

## Discussion

There are more than 800 species of scleractinian corals in the world (Veron 2000). There are discrepancies between conventional taxonomy and molecular data (Fukami et al. 2004). To promote understanding of coral genetics, we have been trying to accumulate molecular cytogenetic data for scleractinian corals. So far, we have published two molecular cytogenetic reports with FISH using the scleractinian corals, *Acropora solitaryensis* (Acroporidae) and *Echinophyllia aspera* (Pectiniidae) (Taguchi et al. 2013, 2014), which are both distributed on the western sea coast of Japan (Wallace 1999). The present study is the third molecular cytogenetic report using *Coelastrea aspera* belonging to the third family Merulinidae.

We carried out G-banding to establish the *Coelastrea aspera* karyotype using embryos. It is difficult in general to obtain a high quality G-banding in invertebrate chromosomes due to the relatively small chromosome sizes and insufficient digestion by trypsin. In this coral, we could produce a G-banding pattern on chromosomes by trypsin-treatment, although it was not fully satisfactory (Fig. 3). By G-banding, we could arrange each chromosome in decreasing order of its length and identify chromosome 11 with an hsr, the presence of which we have already predicted in a previous study through FISH analysis in two corals (*Acropora* and *Echinophyllia*). Visualization of rRNA (28S) gene and telomere sequences by FISH mapping revealed that the rDNA gene locus was on the terminal end of a long arm of chromosome 11, and the telomere sequences were quite similar to humans (TTAGGG)n because we used a human telomere probe as suggested by molecular data (Zielke and Bodnar 2010) and our previous studies (Taguchi et al. 2013, 2014). An hsr was composed of rRNA genes because FISH signals of the rDNA probe were highlighted. It is of interest that large signals seen in the elongated chromosome 11 were confirmed, by G-banding, to be on an hsr which is sometimes seen in human cancer cells as a resultant of oncogene amplification (Takaoka et al. 2012). An hsr was originally found in mammalian cells as the result of a huge number of gene amplifications through drug-selection using methotrexate (Biedler and Spengler 1976). Based on our recent studies of three scleractinian coral species, including *Coelastrea aspera*, an hsr found in the coral chromosomes seemed to be a common and consistent feature in scleractinian corals (Taguchi et al. 2013, 2014). We also observed an hsr-like structure in the two other corals, *Micromussa amakusensis* Veron, 1990 and *Trachyphyllia geoffroyi audouin* Audouin, 1826 (data not shown).

To verify the nature of an hsr composer of rDNA cytogenetically, we tried to visualize NOR proteins using Ag-NOR staining (Bloom and Goodpasture 1976). When we performed Ag-NOR analysis with *Coelastrea aspera*, human metaphase spreads were stained at the same time as a control (about seven pairs of dots appeared on the chromosome stalks of acrocentric chromosomes and several domains seen on human interphase nuclei; Fig. 5B, C). Consequently, five to eight dark dots on the interphase nuclei of the coral were stained with Ag-NORs, whereas no dot seemed to be present on any chromosomes, including hsrs (Fig. 5A). Ag depositions produced by Ag-NOR appear on Ag-NOR related proteins (Trerè et al. 2000). Ag-NOR related proteins remain at metaphase in humans but not in *Coelastrea aspera* (Fig. 5). The fact that no Ag deposition was observed on rRNA gene loci of the coral metaphase chromosomes suggests that Ag-NOR related proteins did not remain on the coral chromosomes, unlike in humans and, as far as known, in all other studied plants and animals (Rodionov 1999).

To elucidate if the hsr of *Coelastrea aspera* is composed only of rDNA or includes some other sequences together with rDNA, we carried out CMD, followed by FISH. We successfully regenerated probes made from the microdissected DNA by DOP-PCR. The FISH experiment confirmed that these probes hybridized on rRNA gene loci and on the terminal end of another chromosome. This suggested that the hsr consists not only of rRNA genes but also some other repeated sequences.

In the previous study (Taguchi et al. 2013), we obtained a probe by PCR using the primers of human satellite III sequences (Fowler et al. 1988) and it gave some specific FISH signals on the chromosomes of the coral *Echinophyllia aspera*. We also revealed that this probe from coral DNA used satellite III primers strongly hybridized to the human chromosome 9 centromere. This indicated that scleractinian coral DNA shared common sequences to not only the human telomere sequence (TTAGGG)n (Zielke and Bodnar 2010) but also human satellite III repetitive sequences. In this study, we confirmed the presence of another human-related Alu-repeated sequence in *Coelastrea aspera*, based on the fact that the Alu-derived probes gave FISH signals to one or two specific portions of some chromosomes. The Alu-repeat family is one of several families consisting of repetitive elements in the human genome. An Alu element is a short stretch of DNA originally characterized by the action of the Alu (*Arthrobacter luteus*) restriction endonuclease (Schmid and Deininger 1975). Alu elements are about 300 base pairs long and are therefore classified as short interspersed elements (SINEs) among the class of repetitive DNA elements. Different kinds of Alu elements occur in large numbers of primates and comprise 11% of the human genome (Kriegs et al. 2007). They have wide-ranging influences on gene expression (Häsler and Strub 2006). We also observed that human satellite DNAs tended to hybridize to the rDNA of *Coelastrea aspera* (data not shown in the results). These suggest that some of the repetitive sequences may be conserved both in corals and humans, as reported in our previous study of *Echinophyllia aspera* (Taguchi et al. 2013). Highly repetitive DNA sequences may become a good tool for identifying phylogenetic positions (Koga et al. 2012). The human-derived probes from satellite III and Alu that we used, would be useful for the detection of specific locations on chromosomes to conduct more precise karyotyping and for criteria classifying coral species.

In conclusion, we have successfully performed chromosome analysis by using conventional banding and molecular cytogenetic techniques, such as G-banding, CMD and FISH. The telomere sequences and rRNA genes could be mapped on the *Coelastrea aspera* chromosomes. Our research suggests that the telomere sequence of *Coelastrea aspera* may be identical to the human telomere sequence. We demonstrated through G-banding that *Coelastrea aspera* has an hsr on chromosome 11. CMD-FISH revealed that the elongated segment of chromosome 11 was an hsr and was occupied by rDNAs and some other sequences. Metaphase spreads with an hsr were seen in more than 50% of cells observed. These new findings would lead to surveys of other scleractinian coral species to find clues in solving difficulties in taxonomy.
